# β-cyclodextrin and succinic acid–driven metabolic enhancement of lipid, phycobiliprotein, and exopolysaccharide production in *Porphyridium purpureum*

**DOI:** 10.1007/s00449-026-03351-5

**Published:** 2026-05-13

**Authors:** Ali Parsaeimehr, Ananda Nanjundaswamy, Giovanni Antonio Lutzu, Alessandro Concas

**Affiliations:** 1 Department of Biology and Microbiology, College of Natural Sciences, South Dakota State University, Brookings, SD USA; 2 Teregroup Srl, via David Livingstone 37, Modena, 41122 MO Italy; 3 Department of Mechanical, Chemical and Materials Engineering, University of Cagliari, Piazza d’Armi, Cagliari, 09123 CA Italy; 4 Interdepartmental Center of Environmental Science and Engineering (CINSA), University of Cagliari, via San Giorgio 12, Cagliari, 09124 CA Italy

**Keywords:** *Porphyridium purpureum*, High-value metabolites, Metabolic modulation, Algal biorefinery, Photoautotrophic cultivation, Sustainable bio-based production

## Abstract

Enhancing the production of high-value metabolites in microalgae is essential for sustainable bioproducts and biofuels. In this study, *Porphyridium purpureum* cultures were supplemented with β-cyclodextrin (β-CD) and succinic acid (SA) to evaluate their effects on biomass, lipid accumulation, fatty acid profiles, phycobiliproteins, and exopolysaccharides. Moderate β-CD concentrations (0.25–0.50 g L^− 1^) significantly increased biomass (2.98 ± 0.12 g L^− 1^), lipid content (277.00 ± 5.56 mg g^− 1^), total fatty acids, and arachidonic acid yield (70.60 ± 5.70 mg L^− 1^) compared to the control. SA supplementation (0.30–0.60 g L^− 1^) selectively enhanced long-chain polyunsaturated fatty acids accumulation compared with the control treatment, with arachidonic acid reaching 45.89 ± 5.70 mg L^− 1^. Both supplements also elevated phycobiliproteins and exopolysaccharides, with phycoerythrin being the dominant phycobiliprotein, reaching 146.88 ± 12.40 mg L^− 1^ under 0.50 g L^− 1^ β-CD and 147.00 ± 7.50 mg L^− 1^ under 0.60 g L^− 1^ SA. Excessive levels of either supplement, however, negatively affected growth and metabolite productivity. These findings demonstrate that controlled β-CD and SA supplementation can efficiently improve biomass and the accumulation of lipids, long-chain PUFAs, PBPs, and EPS in *P. purpureum*, offering a practical strategy for industrial-scale production of high-value algal compounds.

**Supplementary Information:**

The online version contains supplementary material available at 10.1007/s00449-026-03351-5.

## Introduction

Microalgae are photosynthetic microorganisms with great potential for sustainable production of high-value molecules, such as lipids, phycobiliproteins (PBPs), and polysaccharides [1]. Among them *Porphyridium purpureum* is a unicellular red microalga widely recognized for its ability to produce several high-value metabolites with important biotechnological applications, due to its unique physiological and biochemical characteristics [2, 3]. Among these compounds, PBPs, particularly R-phycoerythrin (PE), represent one of the most commercially valuable pigments used in food colorants, cosmetics, and fluorescent labeling technologies [4]. In addition, *Porphyridium* species accumulates lipids rich in polyunsaturated fatty acids (PUFAs) including arachidonic acid (ARA) and eicosapentaenoic acid (EPA), which have nutritional and pharmaceutical relevance [5]. Another distinctive feature of this genus is the production of extracellular polysaccharides (EPS), which exhibit antioxidant, antiviral, and immunomodulatory properties and have attracted increasing interest for biomedical and industrial applications [6]. Because of this unique metabolic profile and its capacity to simultaneously synthesize multiple valuable compounds, *P. purpureum* has become an attractive model organism for studies aimed at improving microalgal productivity and metabolite accumulation [7, 8]. Despite this considerable metabolic potential, industrial use of *P. purpureum* is hindered by low growth rates and poor yields of valuable metabolites under conventional photoautotrophic cultivation systems, such as laboratory-scale shake-flask cultures or controlled photobioreactors operated under standard nutrient and light conditions [9]. To address this concern, chemical modulation has been proposed as a promising strategy to overcome these limitations [10–12].

Among chemical modulators, cyclodextrins (CDs) have been of interest as useful metabolic enhancers. CDs are known as cyclic oligosaccharides consisting of glucose subunits connected by α−1,4-glycosidic bonds that form a hydrophilic outer surface and a hydrophobic cavity. This unique molecular structure allows CDs to form inclusion complexes with hydrophobic or amphiphilic molecules, which leads to increasing solubility, stability, and bioavailability of intracellular metabolites [13, 14]. In microalgae, CDs can induce extracellular release of secondary metabolites, stabilize pigments such as PBPs, and modulate key biosynthetic pathways via their role as carriers or elicitors. In addition, CDs’ ability to interact with a wide range of molecules set them extremely effective in the enhancement of simultaneous production of multiple metabolites [15]. Among the different CD types, β-cyclodextrin (β-CD) is particularly notable due to its optimal cavity size, high water solubility, and low toxicity, making it one of the most ideal candidates for metabolic modulation and metabolite extraction in microalgal systems [16]. Various metabolic modulation strategies have been explored to enhance metabolite production in microalgae. For instance, stress induction through high-light exposure has been widely used to stimulate carotenoid accumulation in *Haematococcus pluvialis*, leading to increased astaxanthin production [17]. Similarly, metabolic and cultivation strategies have been applied to improve lipid accumulation in species such as *Chromochloris zofingensis* [18] and *Nannochloropsis* sp [19]. for biofuel applications. In cyanobacteria such as *Limnospira* (*Spirulina*) *platensis*, environmental and nutritional modulation has been used to enhance phycocyanin production [20], while chemical elicitors have been reported to stimulate secondary metabolite biosynthesis in green microalgae such as *Chlamydomonas reinhardtii* [21]. However, research on β-CD-mediated metabolic enhancement in *P. purpureum* remains limited, and the underlying mechanisms are still unclear.

Meanwhile, succinic acid (SA), a central intermediate in the tricarboxylic acid (TCA) cycle, is another promising molecule for metabolic modulation microalgae [22]. By contributing to cellular energy metabolism and providing key carbon skeletons for biosynthetic pathways, SA can influence both growth and metabolite accumulation [23]. In microalgae, exogenous SA has been reported to enhance biomass production, stimulate lipid biosynthesis, and improve photosynthetic efficiency by modulating the balance between energy production and metabolite flux [24, 25]. Evidence from plant and algal biotechnology suggests that both β-CD and SA can function as metabolic modulators in *P. purpureum.* β-CD have been widely used to enhance the production and extracellular release of secondary metabolites, by forming inclusion complexes with hydrophobic molecules and improving their stability and transport in biological systems [21, 26]. Similarly, SA is a key intermediate of the tricarboxylic acid (TCA) cycle and can influence cellular carbon metabolism and biosynthetic pathways related to lipid and pigment formation in microalgae [27]. Their individual contributions to enhancing metabolite production have not been thoroughly studied, leaving gaps in strategies to maximize the production of lipids, PBPs, and EPS [8]. Although these compounds have shown promising metabolic effects in laboratory-scale studies by stimulating intracellular metabolic fluxes and improving energy balance [23], their economic feasibility for large-scale cultivation depends on process optimization, additive dosage, and downstream recovery efficiency.

Understanding the influence of each molecule provides a foundation for rational chemical modulation of microalgal metabolism. In this study, we investigated the influence of β-CD and SA on *P. purpureum*, with the following specific objectives: (i) evaluate their impact on biomass accumulation and growth, (ii) changes in lipid content and fatty acids (FA) composition, (iii) assess enhancement of PBPs production, and (iv) determine effects on EPS synthesis. By addressing these objectives, this work aims to provide a practical framework for chemical modulation in red microalgae and contribute to the development of efficient and scalable strategies for sustainable production of high-value metabolites.

Unlike previously reported enhancement strategies that focus on single product classes (e.g., lipid-only or pigment-only improvements) or rely on mixotrophy/organic carbon feeding, this work systematically compares two chemically distinct modulators (β-CD and SA) across dose ranges in a red microalga under photoautotrophic cultivation. The aim is to demonstrate concentration-dependent “optimal windows” and how β-CD and SA preferentially boost phycoerythrin (PE), EPS, and long-chain polyunsaturated fatty acids (PUFAs) production. The study therefore advances existing knowledge by providing a multi-output modulation map for *P. purpureum* and a practical, scalable screening framework to guide additive selection based on the desired product portfolio.

The simultaneous enhancement of lipids, PE-rich PBPs, and EPS is relevant to a multi-product microalgal biorefinery model in which high-value, water-soluble fractions (PE/PBPs and EPS) can be recovered first via aqueous processing, followed by lipid extraction from the residual biomass for PUFA or biofuel-oriented streams. Such a cascade can improve resource efficiency by valorizing multiple product fractions from the same cultivation batch and reducing residual waste, thereby strengthening the industrial relevance of metabolic modulation strategies.

This approach is aligned with sustainability goals by supporting bio-based production of functional ingredients, improving resource efficiency through fractionation of multiple co-products, and potentially reducing the environmental burden per unit product by increasing value density per cultivation volume. In this context, chemical modulation under photoautotrophy may contribute to broader sustainability targets such as responsible production, industrial innovation, and climate action through more efficient phototrophic bioprocessing.

## Materials and methods

### Cultivation and maintenance of *P. purpureum*

The marine unicellular red microalga strain *Porphyridium purpureum* UTEX LB2757 was obtained from the Culture Collection of Algae at the University of Texas at Austin, USA. *P. purpureum* was cultured in Guillard’s f/2 medium prepared with filtered artificial seawater adjusted to a salinity of 30 ppt (≈ 30 g kg⁻¹). The composition of the artificial seawater-based medium included macronutrients, trace metals, and vitamins according to the formulation originally described by Guillard and Ryther [28]. Specifically, the culture medium contained 75 mg L⁻¹ NaNO₃ and 5 mg L⁻¹ NaH₂PO₄·H₂O as nitrogen and phosphorous sources. Trace metals and vitamins were added as follows: FeCl₃·6 H₂O (3.15 g L⁻¹), Na₂EDTA·2 H₂O (4.36 g L⁻¹), CuSO₄·5 H₂O (0.01 g L⁻¹), Na₂MoO₄·2 H₂O (0.01 g L⁻¹), ZnSO₄·7 H₂O (0.022 g L⁻¹), CoCl₂·6 H₂O (0.01 g L⁻¹), MnCl₂·4 H₂O (0.18 g L⁻¹), thiamine HCl (200 mg L⁻¹), biotin (1 mg L⁻¹), and cyanocobalamin (1 mg L⁻¹). All media were sterilized by autoclaving at 121 °C for 20 min, except for vitamin solutions, which were filter-sterilized (0.22 μm) and added after cooling. Cultures were maintained in 250 mL Erlenmeyer flasks containing 150 mL of f/2 medium and inoculated with exponentially growing *P. purpureum* cultures at an initial optical density (OD₆₈₀) of 0.1. The inoculum consisted of actively growing pre-cultures maintained in the same f/2 medium under identical cultivation conditions for approximately 5–7 days, corresponding to the exponential growth phase. The inoculum was transferred as a well-mixed cell suspension rather than as concentrated cells to ensure uniform distribution of biomass in the experimental cultures. The flasks, incubated at 25 ± 1 °C under continuous illumination of 100 µmol photons m⁻² s⁻¹, were incubated under continuous agitation on an orbital shaker to ensure homogeneous mixing and adequate gas exchange throughout the cultivation period. The cultivation under batch conditions lasted 20 days and the stationary phase corresponded to the day 20 under these conditions.

### Cell growth and dry weight determination

*P. purpureum* growth was monitored for 20 consecutive days by measuring culture absorbance (ABS) at 750 nm using a spectrophotometer (Thermo Scientific™ GENESYS™). A calibration curve correlating dried biomass concentration with ABS values was established through regression analysis, enabling biomass estimation directly from ABS reading.

The final dry biomass concentration, $\:{X}_{f}$ (g L^− 1^), was calculated according to the approach of Anyanwu et al. [29]:

1 $$\:{X}_{f}=\frac{{\stackrel{-}{W}}_{f}}{{V}_{s}}$$

where $\:{\stackrel{-}{W}}_{f}$ is the mean dry weight (g) measured from triplicate samples collected at the end of cultivation, and $\:{V}_{s}$ is the culture volume (L) processed for the determination.

The net biomass increase, $\:\varDelta\:X$, was obtained:

2 $$\:\varDelta\:X={X}_{f}-{X}_{0}$$

where $\:{X}_{0}$ and $\:{X}_{f}$ denote the initial and final dry biomass concentrations (g L⁻¹), respectively.

Volumetric biomass productivity, $\:{Q}_{X}$, was then computed according to [29]:

3 $$\:{Q}_{X}=\:\frac{{X}_{f}-{X}_{o}}{{t}_{f}-{t}_{0}}=\frac{\varDelta\:X}{\varDelta\:t}$$

where, $\:{X}_{f}$ and $\:{X}_{0}$ are the same variable previously presented while *Δt* corresponds to 20 days.

Volumetric lipid productivity (Pₗ, mg L⁻¹ day⁻¹) was calculated to estimate the average lipid production during the batch cultivation period.

Lipid productivity was determined according to the following equation:

4 $$\:{P}_{L}=\frac{{X}_{f}\times\:LC}{t}$$

where *X*_*f*_ is the final biomass concentration (g L⁻¹), LC is the lipid content of the biomass (mg g⁻¹ dry weight), and t is the cultivation time (days).

### Preparation and use of exogenous modulators

β-CD and SA were selected as exogenous metabolic modulators to assess their effects on the growth and metabolite composition of *P. purpureum*. Both compounds were obtained from Sigma-Aldrich (St. Louis, MO, USA), and stock solutions were freshly prepared prior to use. 10 g L^− 1^ β-CD stock solution was prepared by dissolving the compound in sterile distilled water (45 °C) with gentle stirring, followed by filtration through a 0.22 μm PES membrane. Similarly, 5 g L^− 1^ succinic acid stock solution was prepared in sterile distilled water and filter sterilized. Both stock solutions were added to f/2 culture medium prior to inoculation. β-CD was tested at final concentrations of 0, 0.25, 0.50, 1.00, and 1.50 g L^− 1^, while SA was tested at 0, 0.12, 0.30, 0.60, and 1.20 g L^− 1^. These concentrations were selected to span a low-to-moderate supplementation range while avoiding excessive medium acidification at higher doses, and to capture potential non-linear dose–response behavior. The series was designed to approximate a semi-log progression (near-doubling steps), with 0.12 g L⁻¹ included as a near-threshold dose and 0.30–1.20 g L⁻¹ covering intermediate-to-high levels to identify an optimal window and potential inhibitory effects. β-CD and SA dose–response experiments were conducted as two independent cultivation series; each series included its own matched control flasks prepared and analyzed in parallel with the corresponding treatments. Each compound was added individually at the initial stage of algal growth. The control treatments received an equal volume of sterile distilled water to ensure consistent dilution among treatments.

### Lipid extraction and transesterification

*P. purpureum* biomass collected at the stationary phase of growth, which was determined by monitoring the stabilization of OD₆₈₀ during batch cultivation. Under the experimental conditions applied in this study, cultures reached the stationary phase at approximately day 20. To ensure comparability among treatments, biomass samples from all cultures were collected on the same day for lipid extraction and FA analysis. The sample was transferred into 50 mL centrifuge tubes, followed by the addition of 4 mL distilled water and 5 mL hydrochloric acid (HCl). Samples were heated in a water bath at 70 °C for 20 min, after which 5 mL ethanol was added, and the mixture was allowed to cool to room temperature. Subsequently, 10 mL diethyl ether was added to each sample, followed by shaking and centrifugation at 4500 rpm for 2 min. The ether layer was collected into a round-bottom flask, and the extraction was repeated three times. Total lipid content (mg g ⁻¹ dry weight) was determined by evaporating the combined ether extracts using a rotary evaporator. The procedure used for lipid extraction is based on a classical solvent extraction method described by Bligh and Dyer [30]. Broadly, it is based on a biphasic extraction system using chloroform, methanol, and water. In this protocol, methanol is first used to disrupt cellular structures and promote lipid solubilization, while chloroform facilitates the transfer of lipids into the organic phase. The subsequent addition of water induces phase separation, allowing lipids to partition into the chloroform layer, which is then recovered for analysis.

For transesterification, the extracted lipids were dissolved in chloroform and transferred to a 1.5 mL glass vial. One milliliter of 1 M sulfuric acid in methanol was added, and the samples were incubated at 100 °C for 1 h. After cooling to room temperature, 500 µL distilled water was added and the mixture was shaken for 2 min.

### Fatty acid profiling

The resulting fatty acid methyl esters (FAMEs) were analyzed by gas chromatography (GC) following established acid-catalyzed transesterification procedures commonly used for microalgal lipid analysis, as described by Christie and Ryckebosch [31, 32]. Briefly, GC analysis was performed using an Agilent 7890 GC system equipped with a flame ionization detector (FID) and a DB-WAX column (30 m × 0.32 mm × 0.50 μm). Methyl undecanoate (C11:0, purity 99%) was used as an internal standard. The oven temperature program consisted of three stages: (i) an initial increase from 50 °C to 150 °C at 10 °C min⁻¹, followed by a 2 min hold; (ii) a ramp to 200 °C at 10 °C min⁻¹ with a 6 min hold; and (iii) a final increase to 230 °C at 10 °C min⁻¹, maintained for 5 min. Nitrogen (N₂) was used as carrier gas at a flow rate of 3 mL min⁻¹. The FID was operated with hydrogen and air flow rates of 30 mL min⁻¹ and 300 mL min⁻¹, respectively. Injector and detector temperatures were set at 280 °C and 300 °C, respectively.

### PBPs quantification

Biomass was collected at the stationary phase (day 20) by centrifugation and the cell pellet was resuspended in 0.2 M sodium phosphate buffer (pH 6.85) at a defined biomass-to-buffer ratio to ensure reproducible extraction. Cell disruption was performed using repeated freeze–thaw cycles by freezing the suspension (e.g., − 80 °C for several hours) followed by thawing (e.g., 30 °C water bath) until fully liquefied; this cycle was repeated three times. After disruption, samples were centrifuged (e.g., 10.000 × g, 10–15 min, 4 °C) and the supernatant was collected as the crude PBP extract and kept on ice/in the dark until analysis. Subsequently, quantification was performed according to the method of Bennett and Bogorad [33], using sodium phosphate buffer as the blank. Finally, the absorbance of the supernatant was measured at 615 and 652 nm, and concentrations (mg mL^− 1^) were calculated using the following equations:

5 $$Phycocyanin{\text{ }}(PC){\text{ }} = {\text{ }}[A615 - (0.474{\text{ }} \times A652)]/5.34$$

6 $$Allophycocyanin{\text{ }}\left( {APC} \right){\text{ }} = {\text{ }}\left[ {A652{\text{ }} - {\text{ }}\left( {0.208{\text{ }} \times {\text{ }}A615} \right)} \right]{\text{ }}/{\text{ }}5.09$$

7 $$\begin{aligned}&Phycoerythrin{\text{ }}\left( {PE} \right){\text{ }} \\&= {\text{ }}\left[ {A662{\text{ }} - {\text{ }}\left( {2.41{\text{ }} \times {\text{ }}PC{\text{ }} - {\text{ }}0.849{\text{ }} \times {\text{ }}APC} \right)} \right]{\text{ }}/{\text{ }}9.52\end{aligned}$$

### Total exopolysaccharide (EPS) extraction and quantification

For EPS extraction, 50 mL of culture harvested at the stationary phase was centrifuged at 5000 × g for 10 min to separate the cells from the medium. At the next step, the cell-free supernatant, containing EPS, was collected for EPS precipitation. In this regard, two volumes of cold 95% ethanol were added and mixed with the supernatant and incubated at 4 °C for 12–16 h to facilitate precipitation. Later, the precipitated EPS was recovered by centrifugation at 10.000 × g for 15 min at 4 °C and subsequently dissolved in a minimal volume of distilled water. 0.5 M NaCl was also added to remove bound proteins, followed by a second ethanol precipitation step. The extraction and precipitation procedure follows commonly applied protocols used for recovering extracellular polysaccharides from microalgal cultures [34].

Finally, the total EPS content was measured by phenol-sulfuric acid colorimetric assay for carbohydrate quantification [35]. In brief, 200 µL of the EPS solution was mixed with 200 µL of 5% phenol, followed by the addition of 1 mL of concentrated sulfuric acid,. The samples were incubated for 10 min at room temperature, and the absorbance was measured at 490 nm using a UV-Vis spectrophotometer (Thermo Scientific™ GENESYS™, Thermo Fisher Scientific, USA). Glucose was also used as a standard to generate a calibration curve, and EPS concentration was calculated using the formula:

8 $$\begin{aligned}&EPS{\text{ }}concentration{\text{ }}\left( {mg{\text{ }}L^{{ - 1}} } \right){\text{ }} \\&= {\text{ }}EPS{\text{ }}production{\text{ }}\left( {mg} \right){\text{ }}/{\text{ }}Volume{\text{ }}of{\text{ }}culture{\text{ }}\left( L \right)\end{aligned}$$

where EPS production (mg) is the amount of EPS measured in the supernatant, and Volume of culture (L) is the total culture volume used for EPS extraction.

EPS measurements and statistical comparisons were performed within each experimental series (β-CD series vs. its control; SA series vs. its control), as controls were not shared between the two independent runs.

### Screening-level cost indicator (supplement cost contribution)

The economic assessment presented in this study was designed as a screening-level cost indicator to evaluate the operating-cost contribution associated with β-CD and SA supplementation relative to the achieved biomass yields. This approach does not constitute a full techno-economic analysis (TEA), as it does not include capital expenditure (CAPEX), utilities, labor, harvesting, downstream processing, or financial assumptions. Instead, it quantifies the incremental supplement cost per unit biomass produced under laboratory-scale batch conditions. Market prices of β-CD and SA were obtained from Sigma-Aldrich (USA) at laboratory-grade pricing and used as conservative cost estimates.

The supplement cost contribution per liter of culture was calculated as:

9 $$\:{C}_{supp,L}={P}_{supp}\times\:{C}_{supp}$$

Where $\:{P}_{supp}$ is the supplement concentration (g L⁻¹) and $\:{C}_{supp}$is the unit cost of the supplement (USD g⁻¹).

The total operating cost per liter of culture was estimated as:

10 $$\:{C}_{tot,L}={C}_{supp,L}+{C}_{medium}$$

where $\:{C}_{medium}$ represents the cost of culture medium per liter (USD L⁻¹).

The supplement-associated cost per gram of biomass produced was then calculated as:

11 $$\:{C}_{biomass}=\frac{{C}_{tot,L}}{{Y}_{biomass}}$$

where $\:{Y}_{biomass}$ is the final biomass concentration (g L⁻¹).

To better evaluate the cost-effectiveness of supplementation, an incremental cost indicator was also calculated:

12 $$\:{C}_{{\Delta\:}}=\frac{{C}_{supp,L}}{{\Delta\:}{Y}_{biomass}}$$

where $\:{\Delta\:}{Y}_{biomass}$ represents the increase in biomass yield relative to the corresponding control.

This simplified analysis provides an estimate of the supplement cost burden per unit biomass at laboratory scale and is intended for comparative purposes only, following common cost-normalization approaches used in microalgal techno-economic assessments [36, 37]. A complete TEA would require pilot-scale productivity data, energy consumption metrics, harvesting efficiency, downstream recovery yields, and discounted cash-flow modeling.

### Statistical analysis

The experiment was conducted using a completely randomized design with three independent replicate cultures for each treatment, and data are presented as the mean ± standard deviation of three replicates. Statistical analyses among treatments were evaluated using a one-way analysis of variance (ANOVA) followed by Tukey’s honestly significant difference (HSD) posy hoc test to determine statistically homogeneous groups at a significance level of *p* < 0.05. All statistical analyses were performed using SAS software.

## Results and discussions

### Growth response of *P. purpureum* to β-CD and SA

**Fig. 1:**
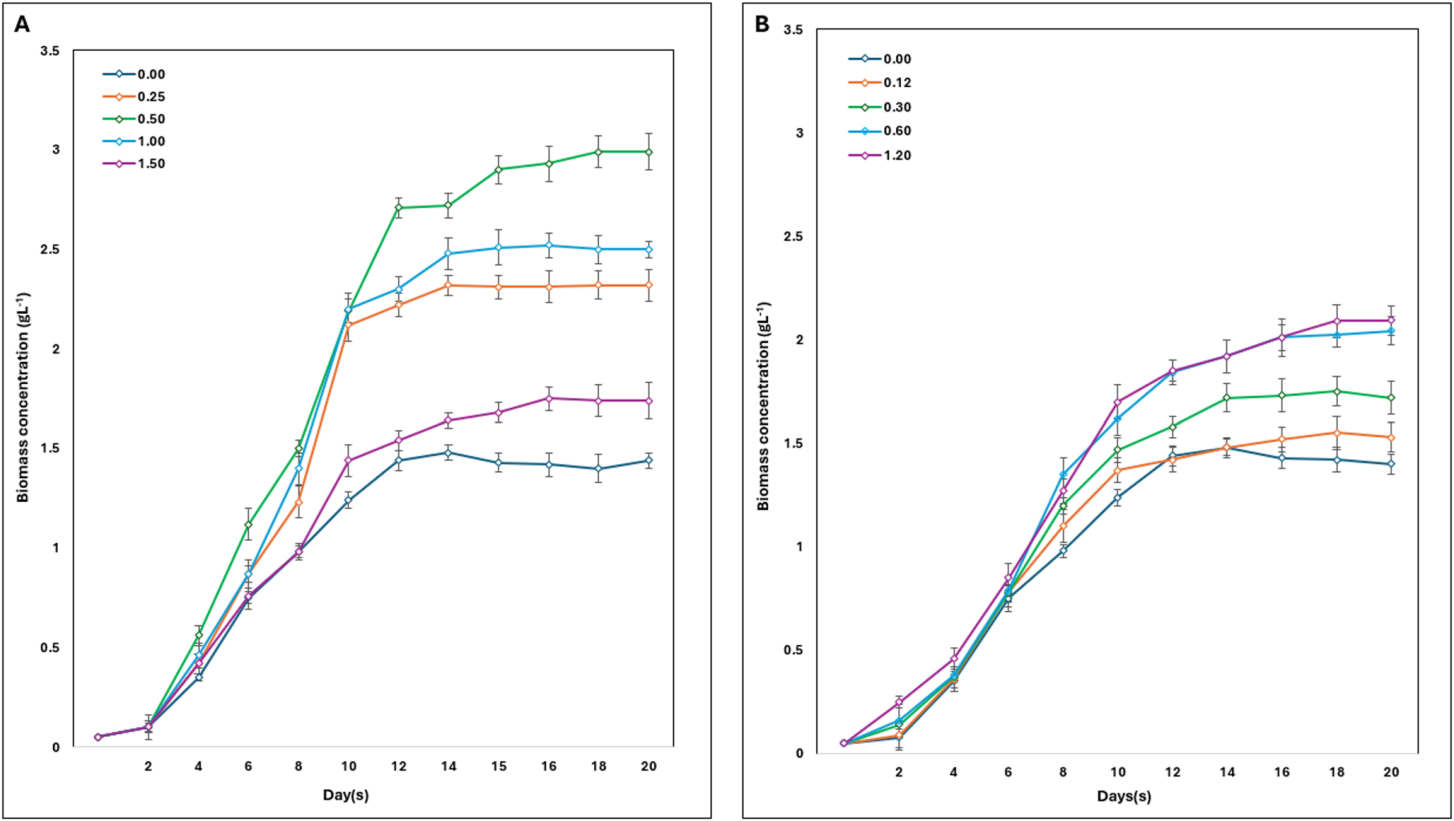
Influence of β-cyclodextrin (**A**) and succinic acid (**B**) on the growth of *P. purpureum*. Note: Biomass concentration (g L^− 1^) was measured over a 20-day cultivation period. Data and error bars represent mean ± SD of three independent replicates

**Fig. 2:**
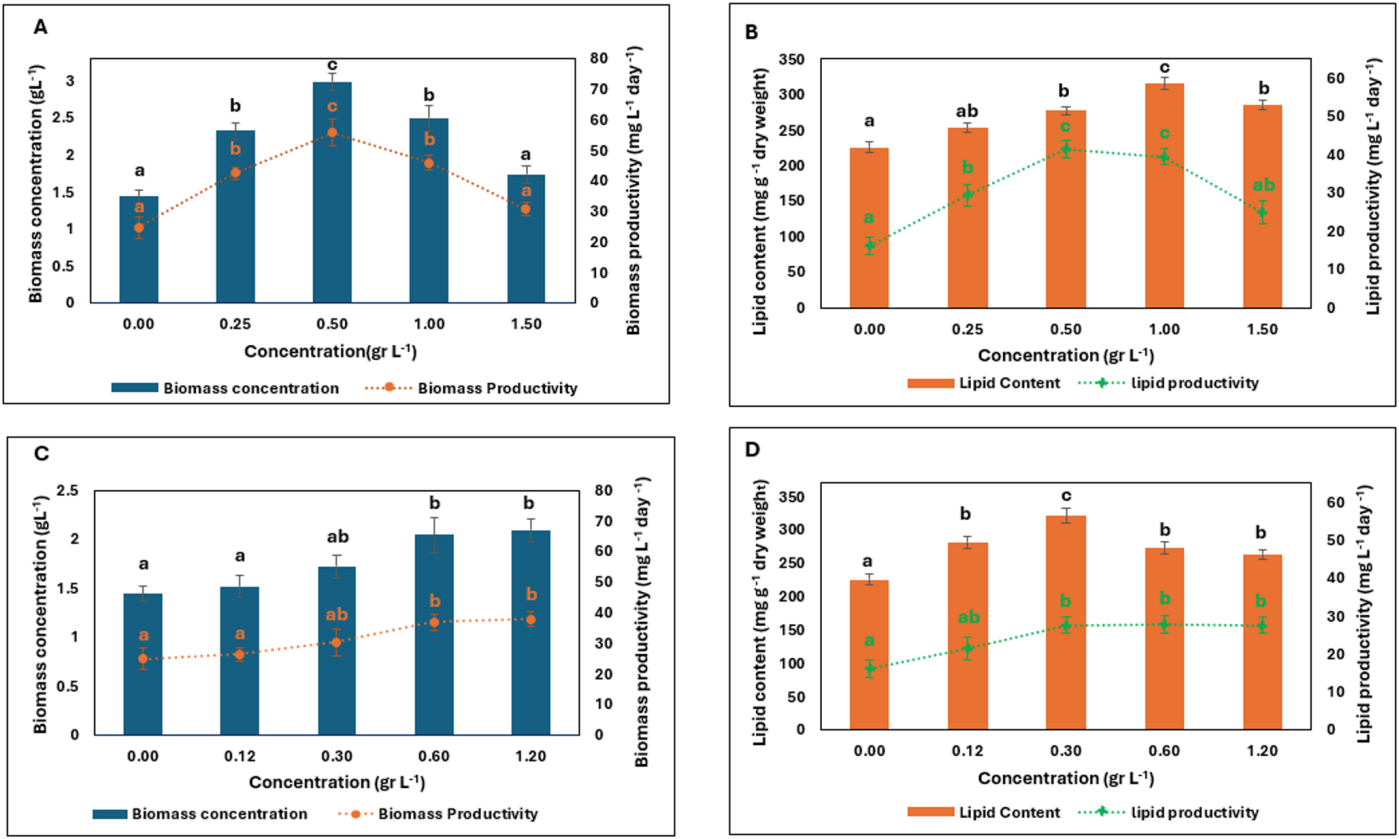
Impact of β-Cyclodextrin (2 **A**-**B**) and Succinic Acid (2 **C**-**D**) on fresh biomass content, biomass productivity, lipid content, and lipid productivity in *Porphyridium purpureum*. Biomass and lipid productivity were calculated based on fresh biomass and lipid content, respectively, with endpoint values from day 20 (stationary phase). Data and error bars represent mean ± SD of three independent replicates, bars labeled with different letters are significantly different from each other at *p* < 0.05

Biomass production was monitored over a 20-day period, and we observed the growth of *P. purpureum* was influenced by the addition of β-CD and SA at the initial stage of cultivation, demonstrating different concentration-dependent effects for both compounds (Figs. 1 and 2). In β-CD treated *P. purpureum* cultures, the highest biomass content (2.98 ± 0.12 g L^− 1^) and the highest biomass productivity of 55.73 ± 4.41 mg L^− 1^ day^− 1^ were obtained at 0.50 g L^− 1^ (Fig. 1A and B, respectively), representing approximately a 2.1-fold increase compared to the control (1.44 ± 0.08 g L^− 1^; 24.80 ± 3.50 mg L^− 1^ day^− 1^). It was observed that increasing concentrations of β-CD beyond the optimal level (1.00–1.50 g L^− 1^) resulted in lower biomass accumulation compared to optimal concentration (0.50 g L⁻¹). However, it is important to note that biomass concentrations at these higher doses remained comparable to or slightly above those of the control cultures, indicating that no severe growth inhibition occurred under the tested conditions. The observed reduction relative to the optimal β-CD concentration therefore suggests a diminishing stimulatory effect rather than a clear toxic response. This trend may reflect concentration-dependent physiological adjustments to β-CD supplementation. Cyclodextrins possess a hydrophilic exterior and a hydrophobic cavity capable of forming inclusion complexes with hydrophobic molecules, which can influence metabolite interactions and extracellular transport processes in biological systems. In the present study, moderate β-CD supplementation (0.25–0.50 g L⁻¹) showed the most favorable responses in terms of biomass and metabolite accumulation, while higher concentrations resulted in lower productivity compared with the optimal range. However, biomass levels at the highest doses remained comparable to the control treatment, suggesting that these concentrations did not exert a strongly inhibitory effect on cell growth. The reduced stimulation observed at higher doses may therefore reflect a diminished efficiency of metabolic modulation rather than direct toxicity. Because parameters such as membrane integrity, osmotic balance, or intracellular metabolite transport were not directly measured, the mechanistic basis of this explanation should be considered hypothetical. Further mechanistic investigations would be required to confirm the underlying cellular responses to high β-CD concentrations.

Similarly, supplementation of *P. purpureum* with SA resulted in a gradual increase in biomass content and productivity, reaching a maximum fresh biomass of 2.09 ± 0.11 g L^− 1^ and a productivity of 37.87 ± 2.20 mg L^− 1^ day^− 1^ at an SA concentration of 1.20 g L^− 1^ (Fig. 2C and D, respectively).

The apparent “optimal” SA concentration depended on the targeted response variable (biomass vs. lipid content vs. PUFA enrichment), highlighting that additive selection and dosing should be guided by the intended product objective. The positive response to SA can be attributed to its role as an intermediate in the tricarboxylic acid (TCA) cycle, providing an additional carbon source and enhancing energy metabolism [38]. The steady increase in growth in SA treatments indicates that *P. purpureum* efficiently assimilates succinate without experiencing inhibitory effects even at the highest tested concentration. A recent study shows that co-culturing *Aurantiochytrium* sp. SW1 with succinate-producing *E. coli* SUC markedly enhances its growth and lipid accumulation (biomass 5.82 g L^− 1^; lipid 1.19 g L^− 1^). Additionally, the exogenous supplementation of succinate demonstrated a considerable stimulatory influence on biomass and lipid accumulation in *Aurantiochytrium* sp. SW1, and notably, at a concentration of 10 g L^− 1^, biomass increased 2.32-fold compared to the control [39].

### β-CD and SA drive increased lipid production and PUFA biosynthesis in *P. purpureum*

The data obtained from 20 days of *P. purpureum* culture showed the supplementation of cultures with either β-CD or SA have significantly influenced also the lipid accumulation and productivity in a dosage-dependent effect (Fig. 2). In this study, lipid productivity values represent the average volumetric productivity calculated over the entire cultivation period rather than a time-resolved lipid production rate. Lipid content increased from 225.00 ± 8.02 mg g^− 1^ dry weight in the control to 277.00 ± 5.56 mg g^− 1^ dry weight at 0.50 g L⁻¹ β-CD, corresponding to an approximately 25% enhancement under this condition. Lipid productivity showed a similar trend, reaching 41.32 ± 2.23 mg L^− 1^ day^− 1^ at 0.50 g L^− 1^, demonstrating 2.6-fold increase compared to the control (16.20 ± 2.40 mg L^− 1^ day^− 1^), while comparable values were also observed at 1.00 g L^− 1^ β-CD. However, by increasing β-CD to 1.50 g L^− 1^, lipid productivity reduced to 24.79 ± 30 mg L^− 1^ day^− 1^, while lipid content (285.33 ± 7.02 g g^− 1^ dry weight) remained higher than the control treatment that might be reflecting stress-induced lipid accumulation in fewer viable cells rather than enhanced overall lipid productivity. Our observations indicate that moderate β-CD levels promote a trade-off between growth and lipid biosynthesis in *P. purpureum*, whereas excessive concentrations may induce cellular stress, shifting metabolism toward storage lipid accumulation at the expense of biomass yield. Previous studies also suggest that the stimulatory effect of β-CD is associated with enhanced membrane permeability, solubilization of hydrophobic intermediates, facilitation of lipid transport and secretion, and potential modulation of carbon partitioning toward fatty acid synthesis [40, 41].

Similarly, moderate SA concentrations (0.12–0.30 g L^− 1^) considerably increased both lipid content and lipid productivity. Lipid content increased from 225.66 ± 8.02 mg g⁻¹ in the control to 281.00 ± 9.61 mg g^− 1^ dry weight at 0.12 g L^− 1^ and reached to a peaked at 321.00 ± 10.54 mg g^− 1^ dry weight with 0.30 g L^− 1^ of SA, accompanied by the highest productivity of 27.56 ± 1.22 mg L^− 1^ day^− 1^ demonstrating 70% increase compared to the control. We also observed that the lipid accumulation decreased slightly at 0.60 and 1.20 g L^− 1^ (272.70 ± 8.74 and 263.30 ± 7.05 mg g^− 1^ dry weight, respectively), while lipid productivity remained relatively stable (~ 27.60–27.80 mg L^− 1^ day^− 1^). These results may be related to the role of SA as an intermediate of the TCA cycle, which can influence carbon metabolism and potentially enhance the availability of precursors for FA biosynthesis [42]. However, higher concentrations may trigger metabolic feedback or redirect carbon toward other energy-demanding processes. While this interpretation is consistent with previous reports on succinate metabolism, confirmation of altered intracellular carbon fluxes would require transcriptomic, proteomic, or metabolic flux analysis. SA is a key intermediate of the TCA cycle and can influence central carbon metabolism and cellular redox balance. Therefore, moderate supplementation may enhance the availability of metabolic intermediates and reducing equivalents that support biosynthetic processes such as fatty acid production [23]. Our results show that 0.50–1.00 g L^− 1^ of β-CD and 0.30 g L^− 1^ of SA represent the best concentrations for highest lipid accumulation and productivity in *P. purpureum*. These findings highlight the potential of these molecules for increasing lipid-rich biomass production for biofuel and bioproduct applications.

**Fig. 3:**
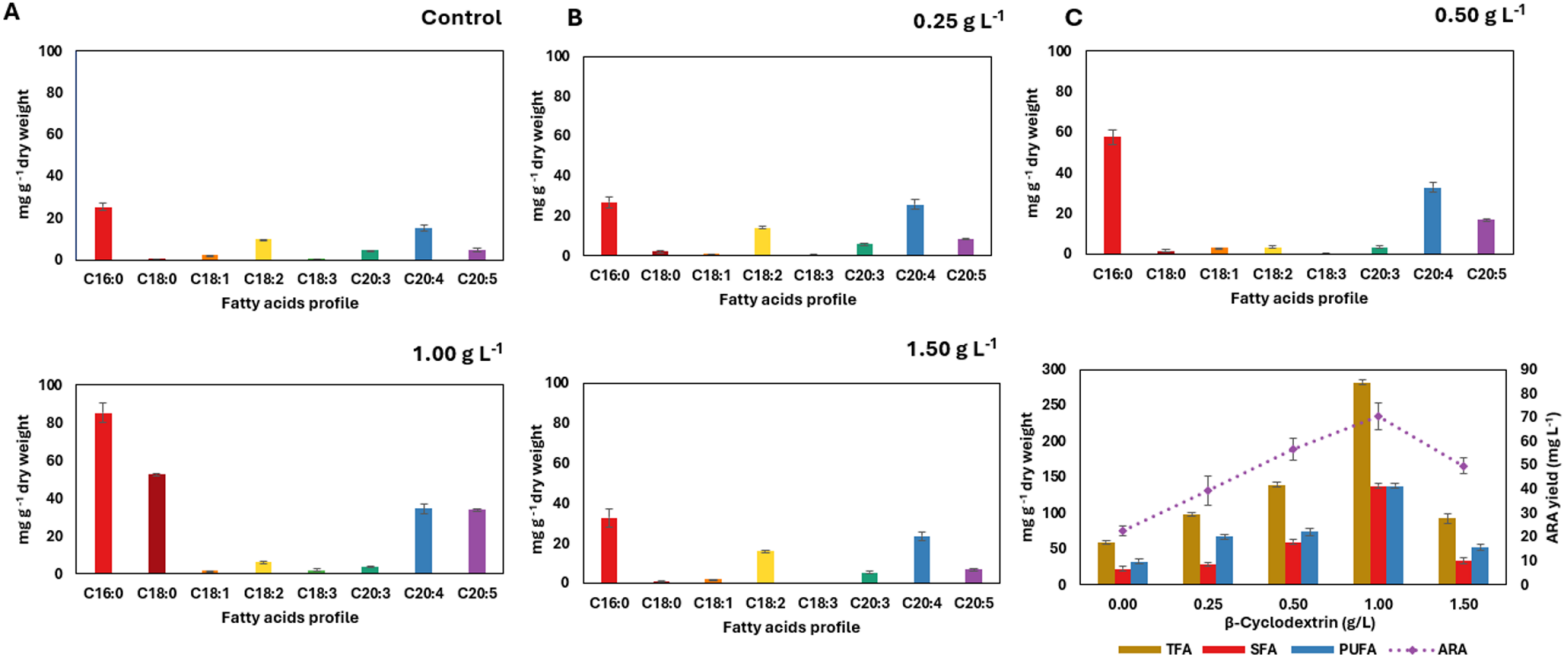
Fatty Acid Profile (3 **A**-**E**) and ARA Production Response (3** F**) to β-Cyclodextrin (β-CD) concentration in *Porphyridium purpureum*. **A** = control, **B** = 0.25 g L^− 1^ β-Cyclodextrin, **C** = 0.50 g L^− 1^ β-Cyclodextrin, **D** = 1.00 g L^− 1^ β-Cyclodextrin, **E** = 1.50 g L^− 1^ β-Cyclodextrin, *TFA* total fatty acids, *SFA * saturated fatty acids, *PUFA *  polyunsaturated fatty acids, *ARA* arachidonic acid. Data and error bars represent mean ± SD of three independent replicates

We also found the FA profile of *P. purpureum* was effectively influenced by β-CD in a concentration-dependent manner (Figs. 3A-E, Table S1). In this regard, total fatty acids (TFA) increased from 59.69 ± 60 mg g^− 1^ dry weight in the control to 282.60 ± 8.40 mg g^− 1^ dry weight at 1.00 g L^− 1^ β-CD, representing 374% of increase. The highest TFA levels were achieved at 0.25–1.00 g L^− 1^ of β-CD concentrations, whereas increasing the concentration to 1.50 g L^− 1^ led to a slight reduction, yielding 92.86 ± 8.40 mg g^− 1^ dry weight, respectively. The ARA yield increased from 22.82 ± 2.06 mg L^− 1^ in control to 39.30 ± 6.20 mg L^− 1^ at 0.25 g L^− 1^, reaching a maximum of 70.6 ± 5.70 mg L^− 1^ at 1.00 g L^− 1^, which represents more than a threefold enhancement compared to the control. Beyond this concentration, the yield declined to 49.70 ± 3.20 mg L^− 1^ at 1.50 g L^− 1^, indicating possible feedback inhibition or diversion of carbon flux into other lipid classes. The observed increase in PUFA, particularly ARA, indicates a shift in FA composition toward more unsaturated species under optimal β-CD supplementation. Such patterns are consistent with reports describing modulation of fatty acid biosynthesis in response to environmental or chemical cues in microalgae. However, since enzymatic activities and gene expression levels were not measured in this study, the specific regulatory mechanisms underlying this shift remain speculative.

Our results also showed the amounts of saturated fatty acids (SFAs) follow a pattern, rising from 22.58 ± 2.30 mg g^− 1^ dry weight in control to 137.73 ± 4.10 mg g^− 1^ dry weight at 1.00 g L^− 1^, then declining at higher concentrations of β-CD. Considerably, poly unsaturated fatty acids (PUFAs) increased from 32.95 ± 2.40 mg g^− 1^ dry weight to 138.12 ± 4.20 mg g^− 1^ dry weight at 1.00 g L^− 1^, reflecting a ~ 319% enhancement (Fig. 3F). Screening the effects of β-CD on the other fatty acids produced by *P. purpureum* revealed that, the palmitic acid (C16:0), arachidonic acid (C20:4), and eicosapentaenoic acid (EPA, C20:5) were accumulated substantially at moderate β-CD levels, while linoleic (C18:2), linolenic (C18:3), and oleic acid (C18:1) demonstrated modest fluctuations. These results suggest that moderate β-CD supplementation (0.25–0.50 g L^− 1^) efficiently increases TFA and the proportion of health-promoting PUFAs, while higher concentrations may induce a saturation effect or metabolic adjustment, emphasizing the importance of optimizing β-CD levels for maximal lipid yield and quality. The observed increase in PUFA production in β-CD treated *P. purpureum* can be described by several connected physiological and biochemical mechanisms. β-CD can form different complexes with hydrophobic molecules, thus increasing the solubility and availability of lipid precursors and membrane-associated compounds. This improved accessibility supports FA elongation and desaturation processes [43]. Furthermore, β-CD interacts with cellular membranes, altering their composition and fluidity, which can further stimulate lipid desaturases activity that is responsible for PUFA biosynthesis [44]. These subtle membrane perturbations may also trigger a metabolic stress response that channels carbon flux toward the formation of unsaturated lipids. Changes in FA saturation patterns may also reflect adjustments in membrane composition under modulator supplementation [45]. Similar trends have been observed in other microalgal species, where stress-induced activation of desaturases led to higher production of C18 unsaturated fatty acids [46]. Overall, β-CD functions not only as a solubilizing and complexing agent but also as a mild elicitor that enhances desaturase activity and directs metabolic flux toward PUFA enrichment in microalgal cells. However, the present study did not quantify dissolved oxygen dynamics or membrane fluidity parameters, and therefore such mechanisms remain hypothetical.

**Fig. 4:**
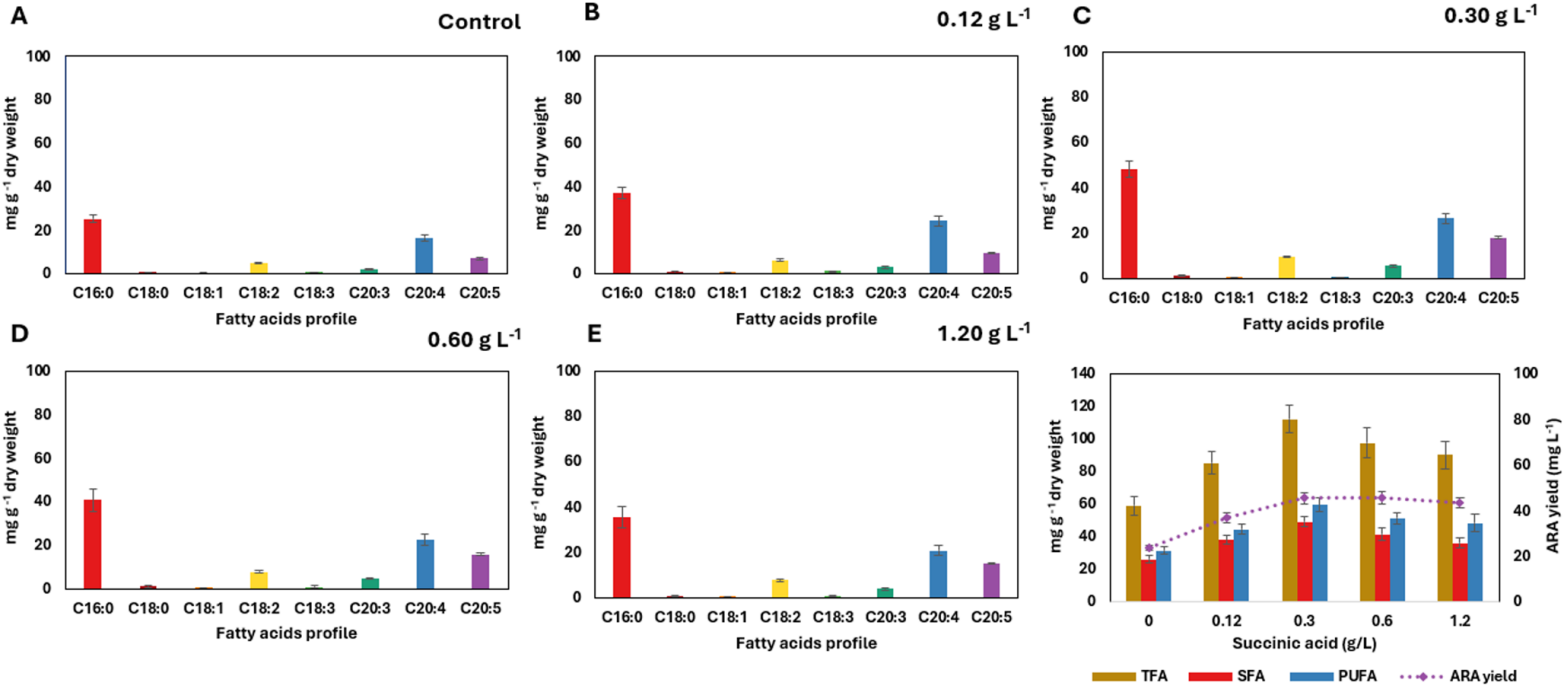
Fatty Acid Profile (4 A-E) and ARA Production Response (4 F) to Succinic Acid (SA) Concentration in *Porphyridium purpureum.* **A** control, **B ** 0.12 g L^− 1^ SA, **C** 0.30 g L^− 1^ SA, **D ** 0.60 g L^− 1^ SA, **E** 1.20 g L^− 1^ SA, *TFA *= total fatty acids, *SFA* = saturated fatty acids, *PUFA * polyunsaturated fatty acids, *ARA * arachidonic acid. Data and error bars represent mean ± SD of three independent replicates

In a similar way to β-CD, supplementation of *P. purpureum* with SA also influenced FA production in a concentration-dependent manner (Figs. 4A-F, Table S2). TFAs increased from 59.06 ± 5.40 mg g^− 1^ dry weight in the control to 112.35 ± 8.40 mg g^− 1^ dry weight at 0.30 g L^− 1^, representing a 90% increase. SFAs followed a similar pattern, increasing from 26.06 ± 2.30 mg g^− 1^ dry weight to 49.18 ± 3.20 mg g^− 1^ dry weight at 0.30 g L^− 1^. Moreover, the production of PUFAs increased from 31.52 ± 2.40 mg g^− 1^ dry weight in the control to 59.60 ± 4.20 mg g^− 1^ dry weight at 0.30 g L^− 1^. The ARA yield increased from 23.77 ± 2.06 mg L^− 1^ in the control to 37.12 ± 6.02 mg L^− 1^ at 0.12 g L^− 1^, reaching its maximum of 45.89 ± 5.70 mg L^− 1^ at 0.60 g L^− 1^, before slightly decreasing to 43.66 ± 3.28 mg L^− 1^ at 1.20 g L^− 1^. This pattern indicates that moderate SA concentrations (0.30–0.60 g L^− 1^) are optimal for ARA accumulation in *P. purpureum* (Fig. 4F).

We also found out that, the key FAs, including palmitic acid (C16:0), arachidonic acid (C20:4), and EPA (C20:5), accumulated most at 0.30–0.60 g L^− 1^, while other FAs such as stearic (C18:0), oleic (C18:1), linoleic (C18:2), and linolenic (C18:3) showed different, concentration-dependent changes (Fig. 4A-E). These findings are supported by the role of SA as a carbon source in enhancing FA production in *P. purpureum*. SA feeds into the TCA cycle, increasing the availability of acetyl-CoA, a direct precursor for FA synthesis, thus raising overall lipid production. Additionally, SA can alter key enzymes involved in lipid biosynthesis, such as acetyl-CoA carboxylase and FA synthases, leading to increase the production of both saturated and PUFAs. However, at higher concentrations, SA may induce metabolic feedback or redirect carbon to other cellular processes, which in return limits the further increases in lipid production [39, 47].

The observed changes in FA composition are particularly relevant from a biotechnological perspective. SFAs contribute to the structural stability of cellular membranes and are also considered suitable feedstocks for biodiesel production due to their oxidative stability and favorable combustion properties. In contrast, PUFAs are of high nutritional and pharmaceutical interest because of their roles in human health and metabolic regulation. Among these compounds, long-chain PUFAs such as ARA and EPA are especially valuable. ARA is widely used in infant nutrition formulations and pharmaceutical products due to its role as a precursor of eicosanoids involved in immune and inflammatory responses. EPA, an omega-3 fatty acid, has well-established health benefits including cardiovascular protection and anti-inflammatory effects, and is commonly incorporated into dietary supplements and functional foods [48]. The ability of *P. purpureum* to produce these high-value fatty acids highlights its potential as a sustainable microbial source of nutritionally important lipids and bioactive compounds.

### PBPs production

**Fig. 5:**
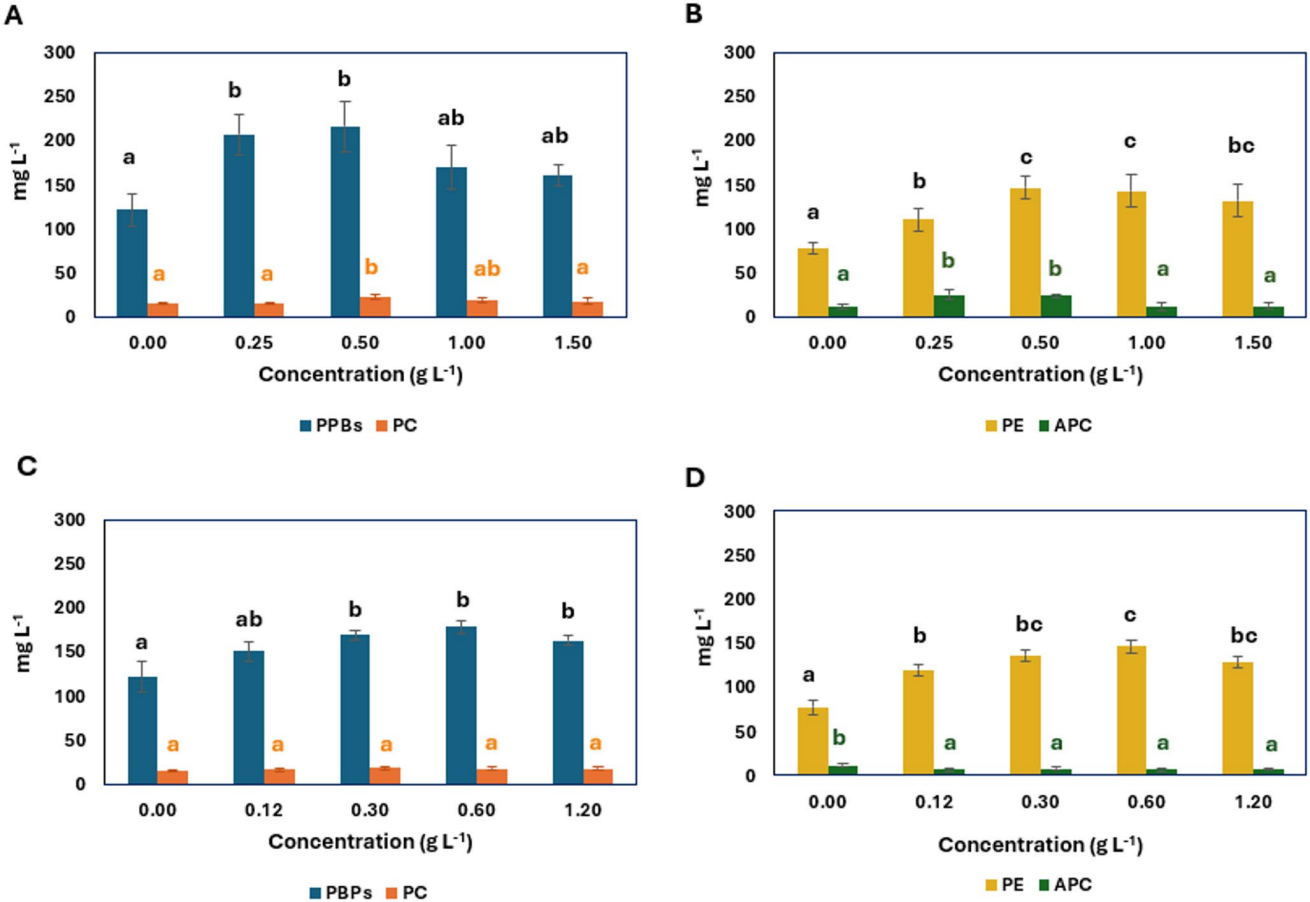
Effect of β-Cyclodextrin (5 A-B) and Succinic Acid (5 C-D) on phycobiliprotein (PBPs), phycocyanin (PC), phycoerythrin (PE), and allophycocyanin (APC) contents in *Porphyridium purpureum.*. Endpoint values are from day 20 (stationary phase). Data and error bars represent mean ± SD of three independent replicates, bars labeled with different letters are significantly different from each other at *p* < 0.05

Total phycobiliproteins (PBPs) content in *P. purpureum* responded significantly to both β-CD and SA supplementation. β-CD enhanced pigment accumulation from 121.14 ± 17.74 mg L^− 1^ in control to 216.60 ± 28.03 mg L^− 1^ at 0.50 g L^− 1^, representing a 78% increase. However, at higher β-CD concentrations, total PBP levels declined slightly to 170.00 ± 25.00 mg L^− 1^, indicating an inhibitory effect beyond the optimal dose (Fig. 5A-B). Similarly, SA supplementation improved total PBPs content from 121.14 ± 17.74 mg L^− 1^ to 178.08 ± 7.60 mg L^− 1^ at 0.30 g L^− 1^, a 39% increase compared to the control while higher concentrations (0.60 g L^− 1^) negatively affected pigment accumulation (Fig. 5C-D). These results suggest that moderate concentrations of both supplements are most effective for enhancing PBPs synthesis, likely due to improved metabolite accessibility and solubilization in β-CD-treated cultures and enhanced energy metabolism through TCA cycle under SA supplementation. We also observed distinct responses in the production of phycocyanin (PC), phycoerythrin (PE), and allophycocyanin (APC). PBPs production in *P. purpureum* was primarily characterized by changes in PE, the dominant and commercially most relevant pigment in this species. *Porphyridium* is widely recognized as a rich source of R-PE, which is used in food colorants, cosmetics, and fluorescent labeling technologies [49, 50]. In the present study, PE was the dominant component, reaching its highest concentration in *P. purpureum* under 0.50 g L^− 1^ β-CD supplementation (146.88 ± 12.40 mg L^− 1^), representing an 88% increase compared to the control (78.08 ± 6.87 mg L^− 1^) (Fig. 5B). A similar enhancement was also observed with 0.60 g L^− 1^ SA treatment, which yielded 147.00 ± 7.50 mg L^− 1^ (Fig. 5D).

Compared to PE, variations in PC and APC were less substantial. PC levels similarly increased to 22.70 ± 2.14 mg L^− 1^ with 0.50 g L^− 1^ β-CD (Fig. 5A) and 18.00 ± 2.00 mg L^− 1^ with 0.30 g L^− 1^ SA (Fig. 5C), while APC reached 24.00 ± 2.20 mg L^− 1^ (β-CD = 0.50 g L^− 1^) and 7.44 ± 1.50 mg L^− 1^ (SA = 0.30 g L^− 1^) (Fig. 5B-D). While PC represents the primary commercial PBP in cyanobacteria such as *Arthrospira*, in red microalgae like *P. purpureum*, PE constitutes the principal pigment fraction and economic target. Similar modulation of PE accumulation under altered light and nutrient conditions has been reported in *P. purpureum* [51], confirming that phycoerythrin biosynthesis is highly responsive to physiological regulation.

Overall, the concentration-dependent enhancement of PBPs observed here, with moderate supplementation of β-CD (0.25–0.50 g L^− 1^) and of SA (0.30–0.60 g L^− 1^), suggests that β-CD and SA act as metabolic modulators rather than simple growth stimulants. However, intracellular regulatory mechanisms governing PBP synthesis were not directly assessed in this study. Therefore, the observed changes should be interpreted as phenotypic responses to chemical modulation, and further transcriptomic or metabolic flux analyses would be required to clarify the underlying biochemical pathways.

The concentration-dependent response observed under β-CD supplementation suggests that moderate levels may enhance pigment accumulation through modulation of extracellular metabolite interactions. Cyclodextrins are known to form inclusion complexes with hydrophobic molecules, which can influence metabolite stability and extracellular dynamics in biological systems [21, 26]. In the present study, β-CD supplementation was associated with increased PE accumulation under optimal concentrations, although intracellular transport processes and membrane-level interactions were not directly assessed. Therefore, the mechanistic basis of this enhancement remains hypothetical.

Similarly, SA supplementation may influence cellular metabolism through its role as a central carbon intermediate. Because succinate participates in primary metabolic pathways [27], moderate supplementation could alter intracellular carbon allocation and biosynthetic capacity. However, parameters such as redox balance, enzyme regulation, and phycobilin precursor availability were not measured here. Consequently, the observed increase in PBP content should be interpreted as a phenotypic response rather than confirmed pathway activation.

Importantly, in *P. purpureum*, PE represents the principal PBP fraction and the most economically relevant pigment [49, 50]. The enhancement of PE under both β-CD and SA supplementation therefore constitutes the most significant outcome of the present study. PC and APC responses were comparatively modest and should be viewed as secondary components of the overall PBP pool.

### Exopolysaccharide (EPS) production

The stimulatory effect of β-CD on EPS production can be linked to its distinctive molecular structure. Cyclodextrins have a hydrophilic outer surface and a hydrophobic internal cavity, constructed from glucopyranose units linked by α−1,4-glycosidic bonds. The hydrophilic exterior ensures solubility in water, while the hydrophobic cavity enables β-CD to form inclusion complexes with a variety of nonpolar or amphiphilic molecules via non-covalent interactions. This dual characteristic allows β-CD to function as a molecular carrier, improving the solubility, stability, and bioavailability of hydrophobic intermediates and signaling molecules in the culture medium. The role of β-CD as molecular carriers and elicitors enhancing the production of bioactive compounds has been extensively described in plant systems [26]. Additionally, β-CD is widely used in plant and algal cell cultures to enhance the synthesis and extracellular release of bioactive secondary metabolites [21]. By forming complexes with secreted products, β-CD can alleviate feedback inhibition and promote the continuous release of these compounds into the medium, thereby stimulating secondary metabolism. In *P. purpureum*, this process likely increases carbon flux towards polysaccharide and pigment biosynthesis, resulting in higher EPS yields. Therefore, the inclusion capacity and elicitor role of β-CD provide a biochemical basis for its concentration-dependent enhancement of EPS production and other bioactive compounds [52].

EPS production in this study increased from 126.00 ± 31.96 mg L^− 1^ in control to 472.00 ± 39.62 mg L^− 1^ at 1.50 g L^− 1^ β-CD, representing a 274% increase. A further increase in the β-CD concentration didn’t show any increase on the EPS levels and had a negative impact on the *P. purpureum* growth. This concentration is comparable to or slightly higher than values reported for *Porphyridium* species under standard batch cultivation, where EPS titers typically range between ~ 80–150 mg L⁻¹ depending on strain and culture conditions [53]. SA supplementation also increased EPS production in *P. purpureum*, although the effect was less compared to β-CD. EPS levels increased from 114.00 ± 20.54 mg L^− 1^ in the control to 174.00 ± 37.14 mg L^− 1^ at 1.20 g L^− 1^ SA, corresponding to a 53% increase (Table 1). The EPS concentrations measured here (≈ 0.11–0.17 g L⁻¹) fall within the lower-to-mid range reported for *Porphyridium* under batch conditions, while higher titers (≥ 1–2.50 g L⁻¹) have been achieved under optimized media and photobioreactor operation [54]. EPS biosynthesis in *Porphyridium* species has been shown to be sensitive to metabolic and environmental modulation, including nutrient and carbon-related factors [53]. Variations in extracellular polysaccharide accumulation under different physiological conditions have also been reported in related *Porphyridium* strains [55], supporting the view that SA-induced metabolic adjustments may contribute to enhanced EPS secretion. However, direct assessment of intracellular carbon partitioning would be required to confirm this mechanism. Our results indicate that both β-CD and SA effectively increased EPS production in *P. purpureum*. β-CD treatment resulted in a pronounced, dose-dependent enhancement, while SA led to moderate but consistent increases at all concentrations tested. These findings suggest that controlled supplementation can optimize EPS yield, supporting its potential use in nutraceutical, pharmaceutical, and biomaterial applications.

**Table 1:** Exopolysaccharide (EPS) yield of *Porphyridium purpureum* under different concentrations of β-cyclodextrin and succinic acid

Compound	Concentration (g L⁻¹)	EPS yield (mg L⁻¹)	% Increase vs. Control
β-Cyclodextrin	0.00	126.00 ± 31.96^a^	–
	0.25	190.00 ± 29.15^b^	50.80%
	0.50	306.00 ± 36.45^c^	142.90%
	1.00	396.00 ± 20.73^d^	214.30%
	1.50	472.00 ± 39.62^e^	274.60%
Succinic acid	0.00	114.00 ± 20.54^a^	–
	0.12	152.00 ± 17.36^b^	33.30%
	0.30	154.00 ± 11.4^b^	35.10%
	0.60	168.00 ± 18.54^b^	47.40%
	1.20	174.00 ± 37.14^b^	52.60%

Values represent mean ± standard deviation of three independent experiments. % Increase is calculated relative to zero-concentration control. Endpoint values are from day 20 (stationary phase). Controls are specific to each independent experimental series (Control for β-CD series; Control for SA series); therefore, control EPS values may differ slightly between series. Data and error bars represent mean ± SD of three independent replicates. Different letters are significantly different from each other at *p* < 0.05

### Economic feasibility of β-CD and SA supplementation

The economic feasibility of supplementing *P. purpureum* cultures with β-CD and SA was evaluated using a screening-level cost indicator based on supplement and medium costs relative to achieved biomass and metabolite titers. This analysis was intended to quantify the operating-cost contribution of supplementation at laboratory scale rather than to provide a full techno-economic analysis (TEA).

Both supplements enhanced overall productivity compared to the control, although their effects differed depending on the target metabolite. Supplementation with 0.50 g L^− 1^ β-CD resulted in a pronounced increase in biomass (2.98 ± 0.12 g L^− 1^) and enhanced accumulation of metabolites, including total lipids (277.00 ± 5.60 mg L^− 1^), PBPs (216.00 ± 28.00 mg L^− 1^), ARA (56.93 ± 4.60 mg L^− 1^), and EPS (306.00 ± 36.50 mg L^− 1^). In comparison, supplementation with 0.60 g L^− 1^ SA selectively stimulated the biosynthesis of long-chain PUFAs, with ARA (45.90 ± 5.70 mg L^− 1^) and EPA (17.30 ± 2.10 mg L^− 1^) representing the highest yields among the tested concentrations (Table 2). When expressed as supplement-associated cost per gram of biomass, the cost analysis revealed that β-CD-supplemented cultures achieved the lowest production cost per gram of biomass ($0.027 g^− 1^) due to significantly improved growth and metabolite output, despite the slightly higher medium cost ($0.082 L^− 1^). Conversely, SA-supplemented cultures exhibited a higher production cost ($0.04 g^− 1^), primarily due to the lower biomass yield. These values should be interpreted as relative supplement cost burdens rather than full production costs. In comparison, increasing the amount of unmodified f/2 medium proportionally raised total biomass and metabolite output but did not improve per-liter yields or the accumulation of high-value compounds, making it a less efficient strategy both biologically and economically.

**Table 2:** Effect of β-cyclodextrin and succinic acid supplementation on biomass, metabolite production, and associated medium costs in *P. purpureum Cultures*

Supplement (g L^− 1^)	Biomass concentration (g L^− 1^)	Total Lipid (mg L^− 1^)	Total PBPs (mg L^− 1^)	Total ALA (mg L^− 1^)	Total ARA (mg L^− 1^)	Total EPA (mg L^− 1^)	EPS (mg L^− 1^)	Medium Cost ($ L^− 1^)	Cost per mg Total Lipid ($ mg^− 1^)	Cost per mg Total PBPs ($ mg^− 1^)	Cost per mg ALA ($ mg^− 1^)	Cost per mg ARA ($ mg^− 1^)	Cost per mg EPA ($ mg^− 1^)	Cost per mg EPS ($ mg^− 1^)	Cost per g Biomass ($ g^− 1^)
Cont.	1.44	225.00	121.40	0.31	23.70	2.50	126.00	0.050	0.0002	0.0004	0.161	0.002	0.020	0.0004	0.034
β-CD (0.50 g L^− 1^)	2.98	277.00	216.00	0.27	70.60	13.40	306.00	0.082	0.0003	0.00038	0.305	0.001	0.006	0.0002	0.027
SA (0.60 g L^− 1^)	2.04	272.00	178.00	0.78	45.90	17.30	168.00	0.081	0.0003	0.00046	0.104	0.001	0.004	0.0004	0.040

*PBPs* phycobiliproteins, *ALA* α-linolenic acid, *ARA* arachidonic acid, *EPA* eicosapentaenoic acid, *EPS* exopolysaccharides

For context, comprehensive TEA studies of algal cultivation consistently identify capital investment, energy consumption, harvesting, and downstream processing as dominant cost drivers, often exceeding medium or additive costs in overall impact [36]. Moreover, for high-value pigments such as PE, purification requirements and product grade strongly influence cost of goods and market price. Therefore, while supplementation improved metabolite titers at laboratory scale, its industrial relevance will ultimately depend on pilot-scale productivity, downstream recovery efficiency, and process integration.

It should also be noted that the cultivation experiments were performed at laboratory scale (250 mL batch cultures), and therefore the reported metabolite titers should be interpreted as proof-of-concept results demonstrating the metabolic potential of supplementation strategies rather than direct indicators of industrial productivity. Translation to commercial systems would require pilot-scale validation under controlled photobioreactor or large-volume cultivation conditions, where productivity, energy demand, harvesting efficiency, and downstream purification strongly influence overall process economics.

It is important to emphasize that the present analysis does not include capital expenditure, utilities, labor, harvesting, extraction, purification, depreciation, or financial modeling. Consequently, the results should be interpreted as preliminary economic indicators intended for comparative assessment of supplementation strategies. A complete TEA would require scale-up productivity data, process energy balances, recovery yields, and discounted cash-flow modeling under defined commercial assumptions.

Within this constrained framework, overall, these results demonstrate that targeted supplementation with β-CD or SA offers a superior approach for optimizing biomass and high-value metabolite productivity in *P. purpureum* cultures. From a multi-product biorefinery standpoint, the differential response observed here β-CD favoring PE/EPS and SA favoring PUFA enrichment, supports a tunable product-portfolio approach in which cultivation and supplementation are selected based on the desired co-product mix. Such co-production can improve resource efficiency by increasing total value recovered per cultivation batch and may reduce the environmental footprint per unit product when integrated with appropriate downstream recovery schemes.

## Conclusion

This study demonstrates that supplementation of *P. purpureum* cultures with β-CD and SA potentially enhances biomass accumulation and stimulate the production of several high-value metabolites, including lipids, long-chain PUFAs, PBPs, and EPS. Moderate β-CD supplementation promoted both growth and lipid biosynthesis, while SA supplementation enhanced lipid and PUFA accumulation, by providing additional carbon flux through the TCA cycle and supporting energy metabolism. Both supplements also increased PBP and EPS levels, although β-CD generally showed pronounced stimulatory effects than SA. Overall, these results highlight the potential of β-CD and SA as cost-effective and efficient metabolic modulators capable of improving the biochemical productivity of *P. purpureum*. The use of targeted supplementation strategies may therefore represent a promising approach to enhance the production of high-value compounds in red microalgae while maintaining photoautotrophic cultivation conditions. Such strategies could contribute to the development of more efficient microalgal bioprocesses for nutraceutical, biotechnological, and bio-based applications.

## Supplementary Information

Below is the link to the electronic supplementary material.

Supplementary Material 1

## Data Availability

No datasets were generated or analysed during the current study.
