# Supplementary material for: β-cyclodextrin and succinic acid–driven metabolic enhancement of lipid, phycobiliprotein, and exopolysaccharide production in Porphyridium purpureum
Source: Bioprocess Biosyst Eng. 2026 May 13;49(6):1677–92. doi: 10.1007/s00449-026-03351-5 (PMC13328284; doi:10.1007/s00449-026-03351-5)
Supplement: Supplementary file 1 — Supplementary Material 1 [file 449_2026_3351_MOESM1_ESM.docx]

**Supplementary Matertial of:**

**β--Cyclodextrin and Succinic Acid–Driven Metabolic Enhancement of Lipid, Phycobiliprotein, and Exopolysaccharide Production in *Porphyridium purpureum***

Ali Parsaeimehr^1*^, Ananda Nanjundaswamy^1^, Giovanni Antonio Lutzu^2^, Alessandro Concas^3,4^

^1^Department of Biology & Microbiology, College of Natural Sciences, South Dakota State University, Brookings, SD USA.

^2^Teregroup Srl, via David Livingstone 37, 41122, Modena, Italy

^3^Department of Mechanical, Chemical and Materials Engineering, University of Cagliari, Piazza d’Armi, 09123 Cagliari, Italy

^4^Interdepartmental Center of Environmental Science and Engineering (CINSA), University of Cagliari, via San Giorgio 12, 09124 Cagliari, Italy

Corresponding author: Ali.Parsaeimehr@sdstate.edu

* Corresponding author:

Ali Parsaeimehr; E-mail: [Ali.Parsaeimehr@sdstate.edu](mailto:Ali.Parsaeimehr@sdstate.edu)

**Table S1. Fatty acid profile response to β-Cyclodextrin concentration**

**in *Porphyridium purpureum***

β-Cyclodextrin concentration

Control 0.25 g L^-1^  0.50 g L^-1^ 1.00 g L^-1^ 1.50 g L^-1^

C16:0 25.34 26.89 57.75 85.17 32.77

C18:0 0.64 2.30 1.83 52.54 1.10

C18:1 2.15 1.01 3.46 1.75 2.11

C18:2 9.74 14.32 3.74 6.15 16.01

C18:3 0.32 0.67 0.38 1.95 0.35

C20:3 4.60 5.83 3.74 4.08 5.48

C20:4 15.29 25.85 33.09 34.54 23.73

C20:5 4.76 8.49 16.97 33.87 6.83

TFA 59.69 98.49 139.98 282.6 92.867

SFA 22.58 29.07 59.59 137.73 33.88

PUFA 32.95 67.27 73.92 138.12 52.86

Note: C16:0 = palmitic acid, C18:0 = stearic acid, C18:1 = oleic acid, C18:2 = linoleic acid, C18:3 = linolenic acid, C20:3 = γ-linolenic acid, C20:4 = arachidonic acid, C20:5 = eicosapentaenoic acid, TFA = total fatty acids, SFA = saturated fatty acids, PUFA = poly unsaturated fatty acids.

**Table S2. Fatty acid profile response to Succinic acid concentration**

**in *Porphyridium purpureum***

Succinic acid concentration

Control 0.12 g L^-1^  0.30 g L^-1^ 0.60 g L^-1^ 1.2 g L^-1^

C16:0 25.34 37.34 47.97 40.67 35.43

C18:0 0.71 0.77 1.21 0.93 0.71

C18:1 0.48 0.62 0.59 0.50 0.51

C18:2 4.97 6.22 9.47 7.68 7.71

C18:3 0.86 1.23 0.27 0.78 0.88

C20:3 2.22 3.14 5.40 4.70 3.85

C20:4 16.50 24.41 26.43 22.46 20.81

C20:5 6.95 9.66 17.97 15.76 15.28

TFA 59.06 85.43 112.35 97.50 90.22

SFA 26.06 38.12 49.18 41.61 36.15

PUFA 31.52 44.69 59.56 51.39 48.55

ARA

Note: C16:0 = palmitic acid, C18:0 = stearic acid, C18:1 = oleic acid, C18:2 = linoleic acid, C18:3 = linolenic acid, C20:3 = γ-linolenic acid, C20:4 = arachidonic acid, C20:5 = eicosapentaenoic acid, TFA = total fatty acids, SFA = saturated fatty acids, PUFA = poly unsaturated fatty acids.
